# PARP-1 Modulation of mTOR Signaling in Response to a DNA Alkylating Agent

**DOI:** 10.1371/journal.pone.0047978

**Published:** 2012-10-24

**Authors:** Chantal Éthier, Maxime Tardif, Laura Arul, Guy G. Poirier

**Affiliations:** 1 Cancer Axis, CHUQ Research Center and Faculty of Medicine, Laval University, Quebec City, Quebec, Canada; 2 Proteomics Platform, CHUQ Research Center and Faculty of Medicine, Laval University, Quebec City, Quebec, Canada; University of Sherbrooke, Canada

## Abstract

Poly(ADP-ribose) polymerase-1 (PARP-1) is widely involved in cell death responses. Depending on the degree of injury and on cell type, PARP activation may lead to autophagy, apoptosis or necrosis. In HEK293 cells exposed to the alkylating agent N-methyl-N’-nitro-N’-nitrosoguanine (MNNG), we show that PARP-1 activation triggers a necrotic cell death response. The massive poly(ADP-ribose) (PAR) synthesis following PARP-1 activation leads to the modulation of mTORC1 pathway. Shortly after MNNG exposure, NAD^+^ and ATP levels decrease, while AMP levels drastically increase. We characterized at the molecular level the consequences of these altered nucleotide levels. First, AMP-activated protein kinase (AMPK) is activated and the mTORC1 pathway is inhibited by the phosphorylation of Raptor, in an attempt to preserve cellular energy. Phosphorylation of the mTORC1 target S6 is decreased as well as the phosphorylation of the mTORC2 component Rictor on Thr1135. Finally, Akt phosphorylation on Ser473 is lost and then, cell death by necrosis occurs. Inhibition of PARP-1 with the potent PARP inhibitor AG14361 prevents all of these events. Moreover, the antioxidant N-acetyl-L-cysteine (NAC) can also abrogate all the signaling events caused by MNNG exposure suggesting that reactive oxygen species (ROS) production is involved in PARP-1 activation and modulation of mTOR signaling. In this study, we show that PARP-1 activation and PAR synthesis affect the energetic status of cells, inhibit the mTORC1 signaling pathway and possibly modulate the mTORC2 complex affecting cell fate. These results provide new evidence that cell death by necrosis is orchestrated by the balance between several signaling pathways, and that PARP-1 and PAR take part in these events.

## Introduction

PARP-1 is a nuclear enzyme involved in various cellular processes including DNA repair, transcription, replication, genomic stability, and cell death [1], [2]. DNA damage resulting from exposure to alkylating agents leads to PARP-1 activation and PAR synthesis [3]. PAR is a branched polymer synthesized from nicotinamide adenine nucleotide (NAD^+^) by PARPs [1]. Most free or protein-associated PAR is rapidly degraded by poly(ADP-ribose) glycohydrolase (PARG) to generate ADP-ribose. It has been recently shown that ADP-ribose is further metabolized very rapidly by NUDIX (**nu**cleoside **di**phosphate linked to another moiety **X**) hydrolases NUDT5 and NUDT9 to generate AMP [4]. AMPK is a sensor of cellular energy that is phosphorylated and activated by the LKB1 tumor suppressor protein kinase under conditions of energy stress that causes high AMP/ATP ratios. AMPK acts to correct the energy imbalance by shutting off ATP consuming processes [5], and one of the major signaling pathways regulated by AMPK is the mammalian target of rapamycin (mTOR) pathway [6].

Autophagy is a basic mechanism to maintain cellular homeostasis and constitutes a survival strategy [7], [8]. However, autophagy has also been linked to programmed cell death [9], [10]. Interdependence between autophagy and apoptosis seems to depend on cell type, the kind of stimulus (strength and duration) as well as on the cellular environment [11]. In normal growth conditions, cells exhibit slow rates of autophagy, because mTOR complex 1 (mTORC1) inhibits this process in response to growth factor signals. mTOR is a large protein kinase of the PIKK (phosphatidylinositol kinase-related kinase) family that exists in two functionally distinct complexes: mTORC1 and mTORC2 [12], [13]. In the mTORC1 complex, mTOR is associated with Raptor, PRAS40 and mLST8, and activation of the complex induces phosphorylation of S6K1/S6K2 and 4E-BP1/4E-BP2, which stimulates transcription, protein synthesis, and cell growth. The mTORC2 complex comprises mTOR, Rictor, SIN1 and mLST8, and the best characterized function of this complex is the phosphorylation of Akt on Ser473 [14]. Interestingly, mTORC2 activates Akt which then stimulates mTORC1, while a feedback loop of mTORC1 on Akt limits Akt signaling [15].

PARP-1 activation is involved in different types of cell death responses. It has been documented that PARP-1 hyperactivation drives the nearly complete depletion of NAD^+^ and ATP pools that leads to cell death by necrosis [16]–[18]. It has also been demonstrated that PARP-1 hyperactivation induces an AIF-dependent apoptosis-like cell death response [19]–[21]. Recently, it has been shown that autophagy might be cytoprotective in response to DNA damaging agents and that PARP-1 activation is involved in the regulation of this process [22]. Based on these findings, we hypothesized that hydrolysis of large amounts of PAR synthesized in response to the alkylating agent MNNG would generate a drastic increase in AMP capable of activating AMPK. Therefore, in this study, we examined the effects of PARP-1 activation by an alkylating agent on the energetic status of cells, on the activation status of AMPK and subsequently on mTORC1 and mTORC2 pathways, which are involved in cell survival and cell death responses.

Our data show that in HEK293 cells, exposure to MNNG leads to NAD^+^ and ATP depletion and also to AMPK activation. We observe an increase in the AMP/ATP ratio, which promotes the phosphorylation of AMPK on Thr172 by the protein kinase LKB1. AMPK activation leads to inhibition of mTORC1, as shown by the phosphorylation of Raptor on Ser792. Furthermore, PARP-1 activation and PAR formation following MNNG exposure possibly affects the mTORC2 complex, as shown by the loss of phosphorylation of the mTORC2 component Rictor on Thr1135. Finally Akt phosphorylation on Ser473 is lost and ultimately cell death by necrosis occurs.

Inhibition of PARP-1 activation with the potent PARP inhibitor AG14361 or in presence of the antioxidant NAC prevents these events. Overall, our results show that PARP-1 activation and massive PAR formation following exposure to an alkylating agent in HEK293 cells affects the signaling pathways regulated by mTOR complexes 1 and 2 and cell fate.

## Results

### PARP-1 Activation by MNNG Induces PAR Formation, NAD^+^ and ATP Depletion, and AMP Synthesis

**Figure 1:**
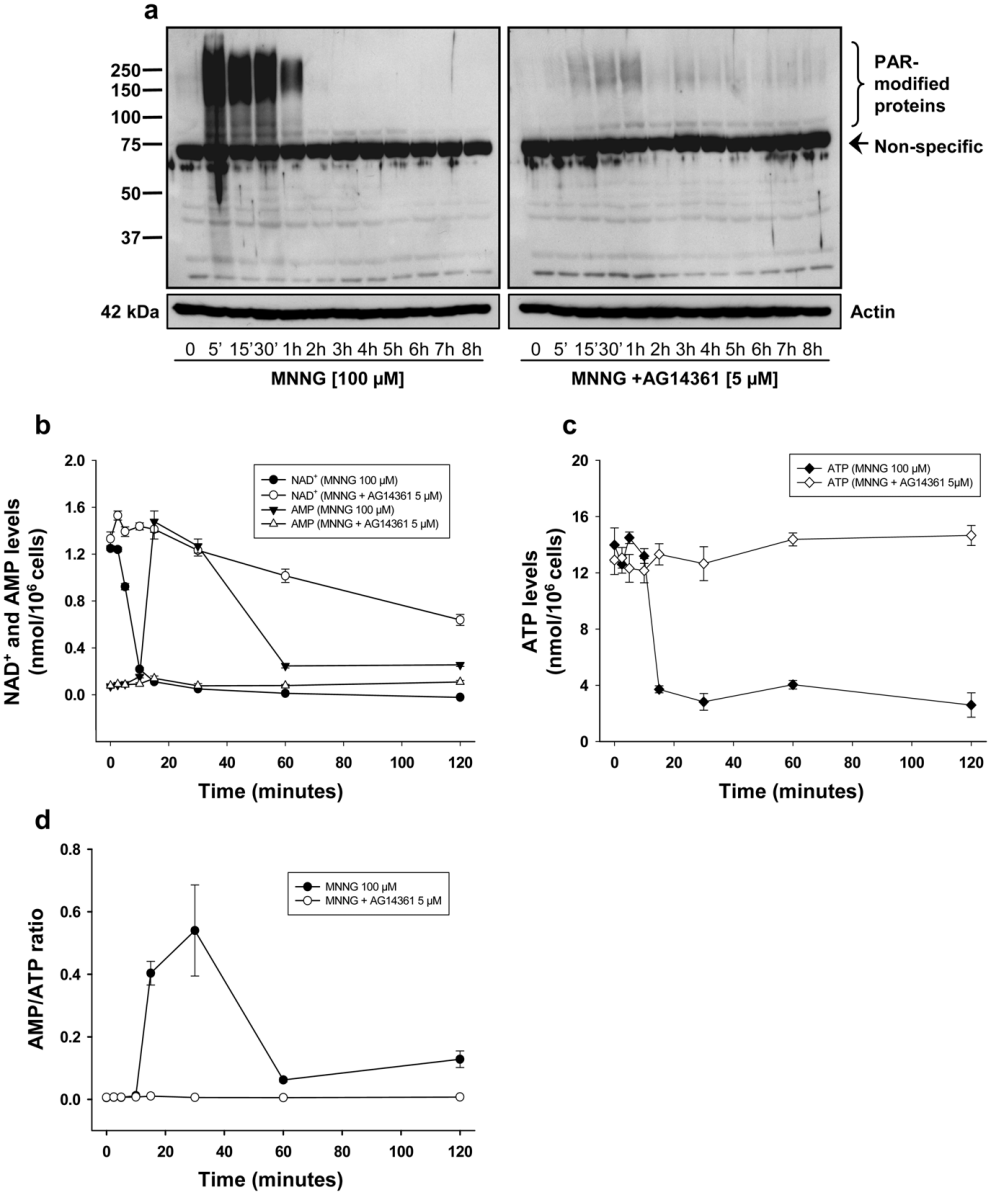
PARP-1 activation following MNNG exposure affects PAR, NAD^+^, AMP, and ATP levels. (a) HEK293 cells were treated with 100 µM MNNG alone (left panel) or in combination with 5 µM of the PARP inhibitor AG14361 (right panel) and monitored for 8 hours. PAR accumulation was detected by western blot using 96-10 antibody. The blots were also probed with actin antibody to show equal loading. (b) NAD^+^ and AMP levels and (c) ATP levels were measured in HEK293 cells treated with 100 µM MNNG alone or in combination with 5 µM AG14361. (d) AMP/ATP ratios were calculated from AMP and ATP values at each time point. NAD^+^, AMP, and ATP levels were measured as described in Materials and Methods. Data are presented as the mean ± standard error of the mean (SEM) of four independent experiments.

PARP-1 activation following MNNG exposure induces massive PAR formation (Fig. 1a, left panel). It has been shown that disproportionate activation of PARP-1 depletes the cellular pools of NAD^+^ and ATP [16], [17], [23], [24]. In order to evaluate the energetic status of cells following PAR formation, we treated HEK293 cells with MNNG with or without the PARP inhibitor AG14361 and measured levels of NAD^+^, ATP, and AMP at different time points after damage. The drastic increase in PAR formation that peaks 5 minutes following MNNG exposure was abolished when cells were treated with AG14361 one hour prior to MNNG exposure (Fig. 1a, right panel). In parallel, we observed a drastic decrease in NAD^+^ levels by 10 minutes (*P*<0.001) and a 20-fold increase in AMP levels by 15 minutes (*P*<0.001) following MNNG exposure (Fig. 1b). Since PAR is synthesized from NAD^+^, PARP-1 overactivation leads to a decrease in NAD^+^ levels. In presence of the PARP inhibitor AG14361, there was no PAR synthesis following MNNG treatment (Fig. 1a, right panel) however, NAD depletion was only partially prevented by PARP-1 inhibition (Fig. 1b), suggesting that another NAD-dependent enzyme is activated by the DNA alkylating agent MNNG. The drastic increase in AMP levels is most likely due to the degradation of PAR by PARG and NUDIX enzymes [4]. We also observed a drastic decrease in ATP levels 15 minutes following MNNG exposure (*P*<0.001) (Fig. 1c). The inhibition of PARP-1 activation by AG14361 prevented ATP loss following MNNG exposure (Fig. 1c). Similarly, AMP levels remained at control levels at all time points when cells were treated with AG14361 before MNNG exposure (Fig. 1b). The variation in ATP and AMP levels following PAR synthesis in response to MNNG exposure was illustrated with the AMP/ATP ratio (Fig. 1d). We observed a significant increase in the AMP/ATP ratio 15 minutes following MNNG exposure, with a peak at 30 minutes (*P*<0.05). At 60 and 120 minutes, we observed a decrease in the AMP/ATP ratio which however remained significantly higher than the AMP/ATP ratio before MNNG exposure (0.062±0.006 and 0.128±0.026 versus 0.005±0.001, *P*<0.001 and *P*<0.03 respectively). We observed no change in the AMP/ATP ratio when cells were treated with AG14361 before MNNG exposure. These results link PARP-1 activation and PAR synthesis to AMP accumulation following exposure to a DNA alkylating agent.

### PARP-1 Activation Induces AMPK Activation and Affects mTORC1/2 Complexes

**Figure 2:**
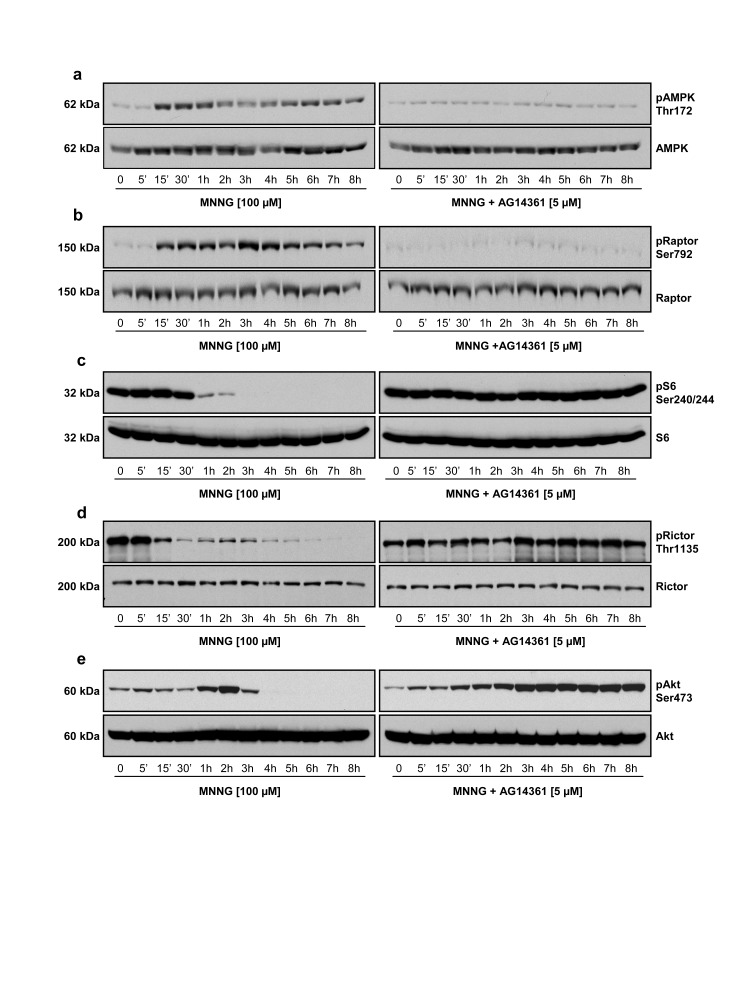
PARP-1 activation is associated with AMPK activation and modulation of mTORC1/2 signaling. HEK293 cells were treated with 100 µM MNNG alone or in combination with 5 µM AG14361. Cell lysates were analyzed by immunoblotting for (a) AMPK phosphorylation on Thr172, (b) Raptor phosphorylation on Ser792, (c) S6 phosphorylation on Ser240/244, (d) Rictor phosphorylation on Thr1135, and (e) Akt phosphorylation on Ser473. Total protein (AMPK, Raptor, S6, Rictor, and Akt) was measured as an internal control (lower panels). Immunoblots are representative of three to four independent experiments.

It is well documented that an increase in AMP/ATP ratio is the key signal to activate AMPK in an attempt to preserve cellular energy [25], [26]. In order to determine whether the increase in AMP levels, caused by PARP-1 activation, leads to the activation of AMPK, we analyzed the phosphorylation levels of AMPK on Thr172 by immunoblotting. We observed a significant activation of AMPK for up to 8 hours, 15 minutes after MNNG exposure in HEK293 cells (Fig. 2a and S1a, left panels). The activation of AMPK is coupled with a 75-fold increase in the cellular AMP/ATP ratio (Fig. 1d). Although the levels of AMP decreased gradually at 60 and 120 minutes (Fig. 1b), the AMP/ATP ratio at 60 and 120 minutes remained significantly higher than the ratio before MNNG exposure (Fig. 1d). Since AMPK is allosterically activated by AMP and AMPK acts in an ultrasensitive manner [27], it is probably sufficient to maintain the activation of the enzyme for up to 8 hours. We also observed concomitant phosphorylation of the mTORC1 component Raptor on Ser792 (Fig. 2b and S1b, left panels), Raptor being a direct substrate of activated AMPK [28]. Then, we observed a drastic decrease in the phosphorylation of the mTORC1 target S6 ribosomal protein, 1 hour following MNNG exposure (Fig. 2c and S1c, left panels). Furthermore, we observed a significant decrease in the phosphorylation of Rictor on Thr1135, which is a key component of the mTORC2 complex (Fig. 2d and S1d, left panels). In cells exposed to AG14361 prior to MNNG, activation of AMPK and phosphorylation of Raptor were abrogated (Fig. 2a and S1a, 2b and S1b, right panels), while phosphorylation of S6 and Rictor was maintained (Fig. 2c and S1c, 2d and S1d, right panels), indicating that these events are specifically triggered by PARP-1 activation. Interestingly, we observed a significant activation of Akt phosphorylation at Ser473 one to three hours after MNNG exposure, followed by a shut-down at 4 hours (Fig. 2e and S1e, left panels), while inhibition of PARP-1 activation and PAR synthesis by AG14361 treatment led to a sustained and significant activation of Akt phosphorylation (Fig. 2e and S1e, right panels). This induction of Akt phosphorylation by PARP inhibitors has been observed previously [29], [30] and might be attributed to the activation of DNA-protein kinase (DNA-PK) after DNA double-stranded breaks (DSB) that persist due to PARP inhibition [31].

In summary, these data suggest that the PARP-1-dependent AMP accumulation following MNNG exposure leads to AMPK activation which affects some components of the mTORC1/2 signaling pathways.

### Effect of PARP-1 Activation in LKB1-deficient HeLa Cell Line

**Figure 3:**
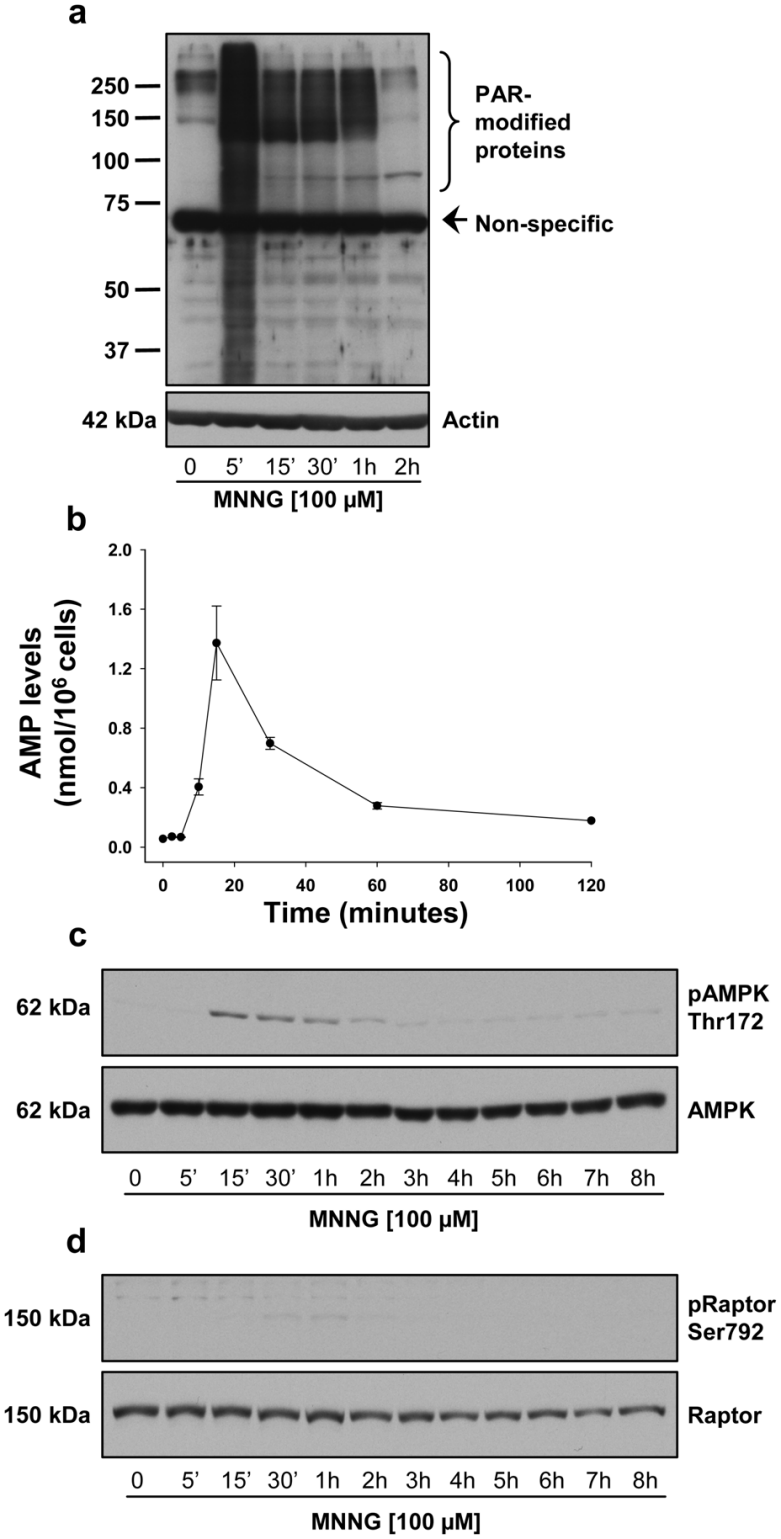
Effect of PARP-1 activation on mTORC1 signaling in HeLa cells. Cells were treated with 100 µM MNNG and monitored for up to 8 hours. (a) PAR accumulation was detected by western blot using 96-10 antibody. The blots were also probed with actin antibody to show equal loading. (b) AMP levels. (c) AMPK phosphorylation on Thr172 (upper panel) and total AMPK protein (lower panel). (d) Raptor phosphorylation on Ser792 (upper panel) and total Raptor protein (lower panel).

The inhibition of mTORC1 after PAR synthesis appears dependent on AMPK activation. To obtain further support of this observation, we examined the same pathway in HeLa cells, which are LKB1-deficient. In HeLa cells, we observed a drastic increase in PAR formation by 5 minutes (Fig. 3a), and a 25-fold increase in AMP levels 15 minutes following MNNG exposure (Fig. 3b). However, only a small and short activation of AMPK (Fig. 3c) was observed, as AMPK was phosphorylated on Thr172 but no phosphorylation of the mTORC1 component and of the AMPK substrate, Raptor, was detected (Fig. 3d). These observations suggest that mTORC1 inhibition in HEK293 cells is linked to the activation of AMPK by the AMP-dependent pathway mediated by LKB1.

### Effect of PARP-1 Activation on Beclin 1 Expression and on the Autophagic Vesicle Marker LC3

**Figure 4:**
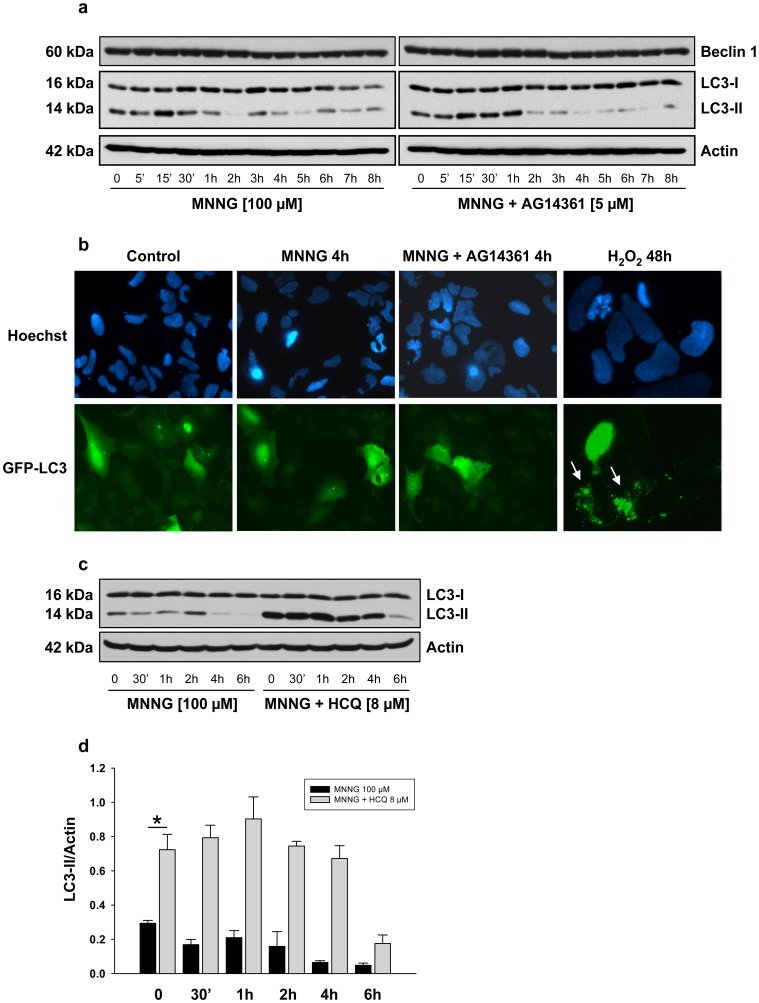
Effect of MNNG exposure on Beclin 1 expression and on the autophagic vesicles marker LC3. HEK293 cells were treated with 100 µM MNNG alone or in combination with 5 µM AG14361 one hour prior to MNNG exposure. (a) Beclin 1 expression and the conversion of LC3-I to LC3-II were detected by immunoblotting. The blots were also probed with actin antibody to show equal loading. (b) Cells were transiently transfected with the GFP-LC3 plasmid for 24 hours and incubated with MNNG alone or in combination with AG14361. Nuclei were stained with Hoechst 33342. The distribution of GFP-LC3 was examined by fluorescence microscopy 4 hours after the start of MNNG exposure and representative cells were photographed. As a positive control for GFP-LC3 redistribution, cells were treated with 1 mM H_2_O_2_ for 48 hours (lower right panel). Punctuated distribution of GFP-LC3 is indicated by the arrows. (c) HEK293 cells were treated with MNNG alone or in combination with the lysosomal protease inhibitor hydrochloroquine sulfate (HCQ) and LC3-II accumulation was detected by immunoblotting. (d) LC3-II/Actin ratios were calculated at each time point. Data are presented as the mean ± standard error of the mean (SEM) of three independent experiments. * *P*<0.005. One way analysis of variance (ANOVA) shows no significant changes in LC3-II accumulation in MNNG+HCQ-treated cells between 0 and 4 hours.

Since we observed that AMPK activation resulted in the suppression of mTORC1 signaling, we verified whether autophagy was subsequently induced following MNNG exposure. The conversion of LC3-I to LC3-II, a marker for autophagic vesicles and autophagy activity [32], was analyzed by immunoblotting. Unexpectedly, we did not observe significant conversion of LC3-I to LC3-II following PARP-1 activation by MNNG exposure (Fig. 4a, left panel). Autophagosome formation is characterized by a punctuated distribution of GFP-LC3. Cells were transiently transfected with GFP-LC3 and subcellular localization was detected by fluorescence microscopy. As shown in Fig. 4b, we did not observe punctuated GFP fluorescence suggesting that autophagy is not induced following MNNG exposure. In contrast, GFP-LC3 was redistributed to produce a punctuate signal 48 hours after H_2_O_2_ treatment as previously described [9] (Fig. 4b, lower right panel). In order to verify whether the autophagic flux is affected by MNNG exposure we measured the amount of LC3-II delivered to lysosomes by comparing the amount of LC3-II in presence and absence of the lysosomal protease inhibitor hydrochloroquine sulfate (HCQ). We observed that HEK293 cells are autophagic competent since there is a significant accumulation of LC3-II in presence of HCQ alone (Fig. 4c, lane MNNG (0) compared to MNNG+HCQ (0), and Fig. 4d, *P*<0.005). However, we did not see further accumulation of LC3-II in presence of MNNG + HCQ from 30 minutes to 4 hours confirming that autophagy is not induced following MNNG exposure (Fig. 4d). Furthermore, we observed a significant decrease in LC3-II accumulation 6 hours following MNNG exposure suggesting that cells became autophagic defective. We also measured the expression of Beclin 1, which is an essential autophagic protein [33], [34] and observed no change in the levels of Beclin 1 following MNNG exposure (Fig. 4a). Therefore, our results suggest that although mTORC1 is inhibited in HEK293 cells following MNNG exposure, autophagy is not induced.

### Oxidative Stress Implication in PARP-1 Modulation of mTOR Signalling

**Figure 5:**
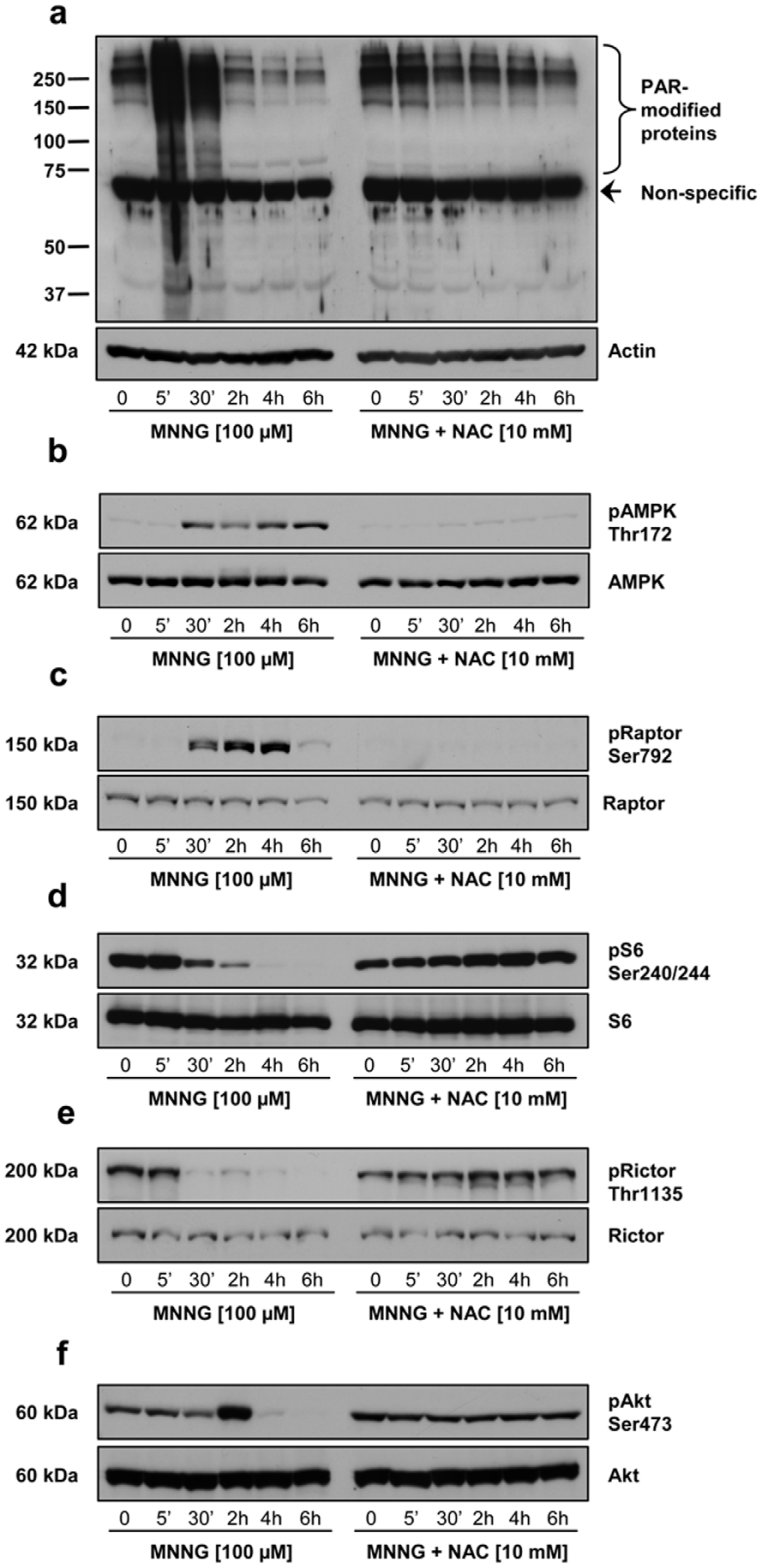
Effect of the antioxidant N-acetyl L-cysteine (NAC) on PARP-1 activation and mTORC1/2 signaling. HEK293 cells were treated with 100 µM MNNG alone or in combination with 10 mM NAC. Cell lysates were analyzed by immunoblotting for (a) PAR accumulation using 96-10 antibody. The blots were also probed with actin antibody to show equal loading. (b) AMPK phosphorylation on Thr172, (c) Raptor phosphorylation on Ser792, (d) S6 phosphorylation on Ser240/244, (e) Rictor phosphorylation on Thr1135, and (f) Akt phosphorylation on Ser473. Total protein (AMPK, Raptor, S6, Rictor, and Akt) was measured as an internal control (lower panels). Immunoblots are representative of three independent experiments.

It has been recently shown that oxidative stress initiates MNNG-induced PARP-1-dependent cell death [35]. In order to evaluate the effect of ROS production on PARP-1 activation, AMPK activation and mTOR modulation, we treated cells with the antioxidant NAC one hour before MNNG exposure. The peak of PAR formation observed at 5 minutes following MNNG exposure was abolished in presence of NAC (Fig. 5a) as well as the modulation of AMPK (Fig. 5b and S2a), Raptor (Fig. 5c and S2b), S6 (Fig. 5d and S2c), Rictor (Fig. 5e and S2d), and Akt (Fig. 5f and S2e) phosphorylation. These results confirm that ROS production triggered by MNNG exposure results in PARP-1 activation and modulation of the mTOR signaling pathway.

### Inhibition of PARP-1 Prevents MNNG-induced Cell Death

**Figure 6:**
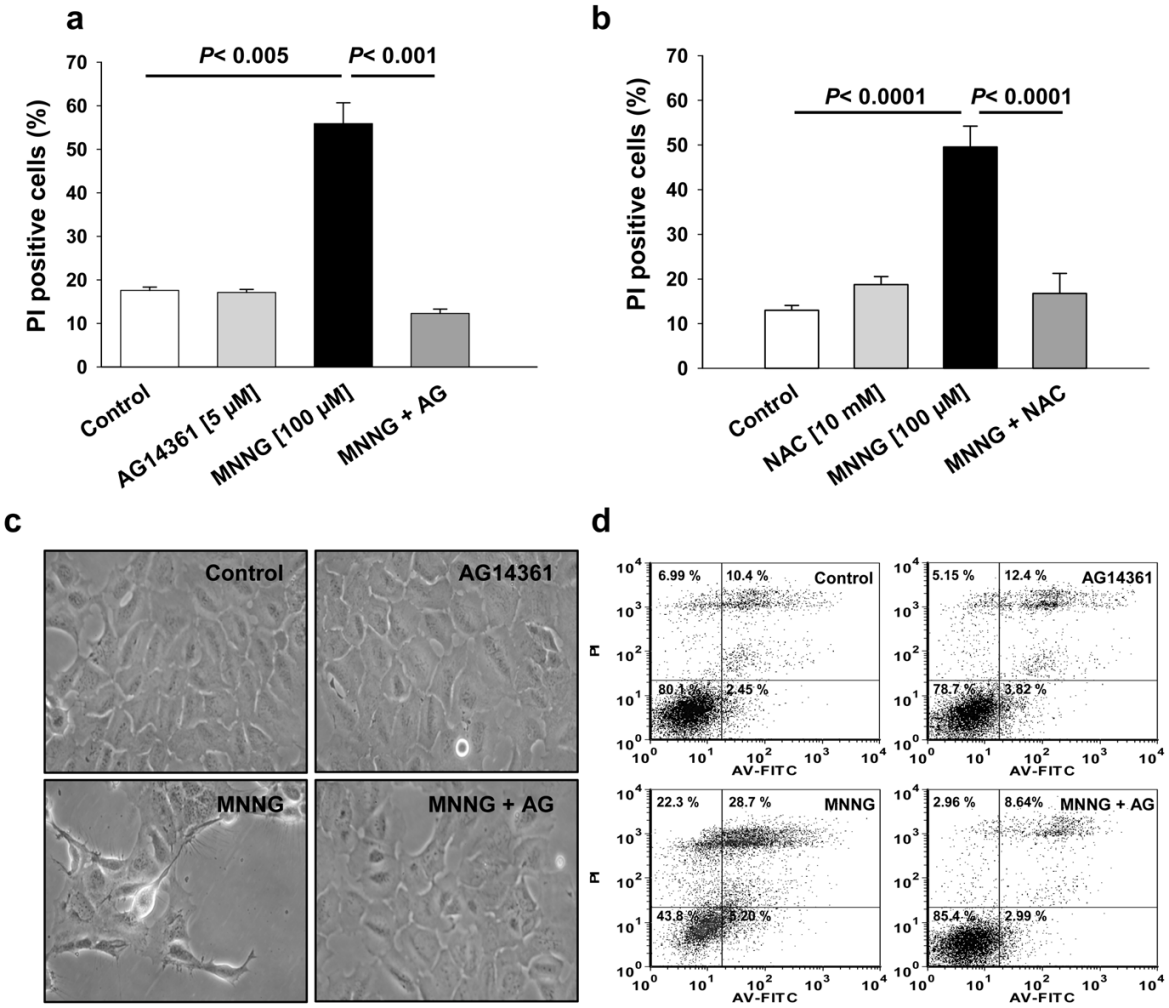
Effect of PARP-1 activation on MNNG-induced cell death. HEK293 cells were treated with 100 µM MNNG alone or in combination with 5 µM AG14361 or 10 mM NAC one hour prior to MNNG exposure. Cell death was evaluated as percentage of PI positive cells 6 hours after MNNG treatment with or without (a) AG14361 or (b) NAC by staining with PI and Annexin-V-FITC coupled with flow cytometry. Data are presented as the mean ± SEM of at least three independent experiments. (c) Phase-contrast images of control cells (upper left panel) and 6 hours after the start of MNNG exposure (lower left panel). HEK293 cells treated with the PARP inhibitor alone (upper right panel) or before MNNG exposure (MNNG + AG, lower right panel) were protected from detachment and were morphologically similar to control cells. (d) Representative data showing cell distribution as evaluated by staining with PI (y axis) and Annexin-V-FITC (x axis) from a population of 10,000 cells.

To evaluate the percentage of cell death induced by MNNG, cells were stained with Annexin-V-FITC and PI and analyzed by flow cytometry 6 hours after MNNG exposure alone or in the presence of AG14361 or NAC. We observed an increase in PI positive cells, 6 hours after MNNG addition, which was significantly reduced when the PARP inhibitor AG14361 was present (55.9±4.8% versus 12.3±1.0%, Fig. 6a and d; *P*<0.001). The number of PI positive cells was also significantly reduced when cells were incubated with the antioxidant, NAC one hour before MNNG exposure compared to MNNG alone (16.8±4.5% versus 49.5±4.7%, Fig. 6b: *P*<0.0001). Morphological examination of cells showed cell detachment without the appearance of apoptotic features, such as membrane blebbing, 6 hours after MNNG addition (Fig. 6c, lower left panel) suggesting that death is rather occurring through necrosis. Cell detachment was prevented in the presence of AG14361 (Fig. 6c, lower right panel), and also in presence of NAC (not shown).

### Type of Cell Death Induced by MNNG Exposure

**Figure 7:**
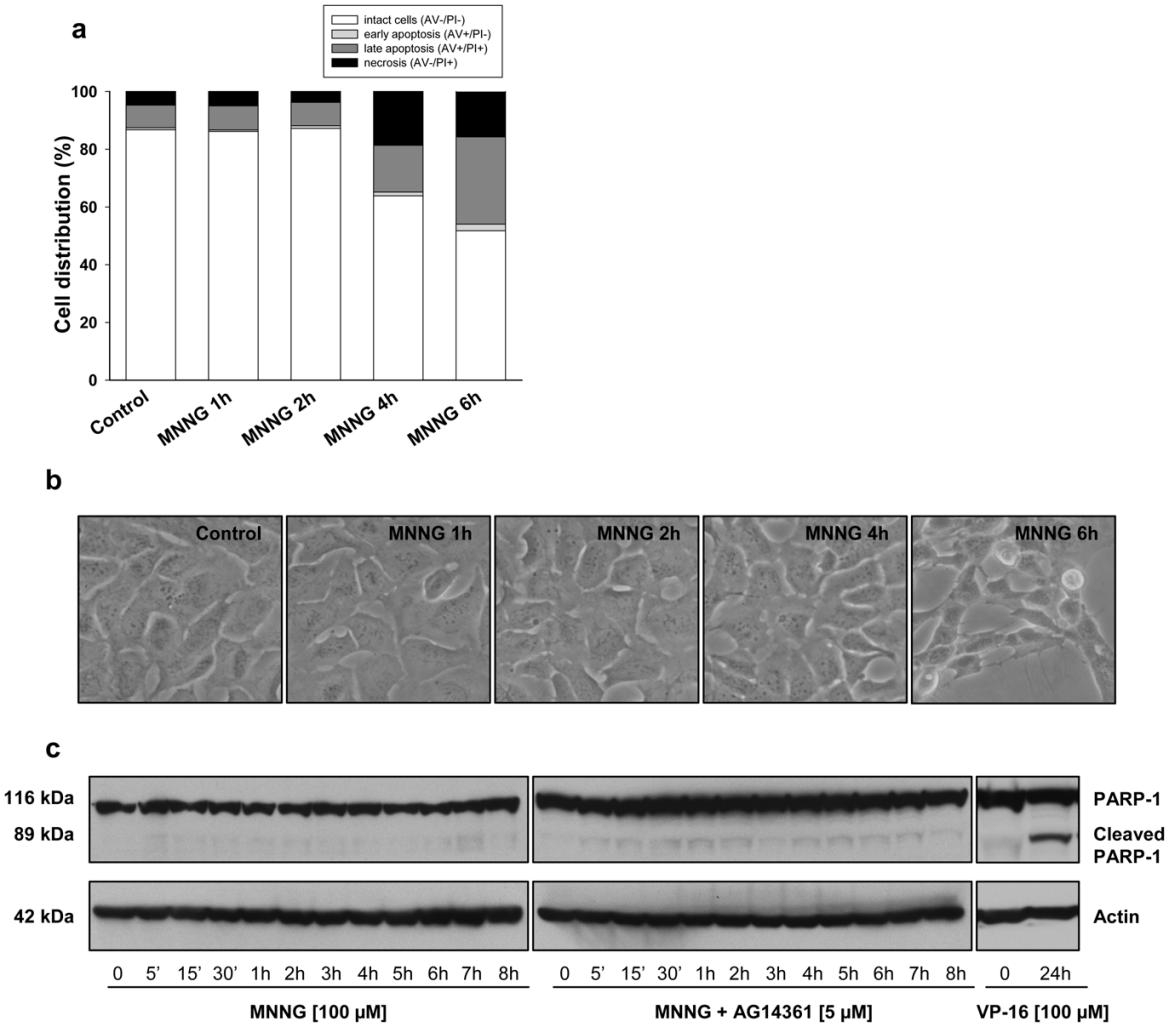
Evaluation of the type of cell death following MNNG exposure (a) Cell distribution at different time points following MNNG exposure by staining with PI and Annexin-V-FITC coupled with flow cytometry. (b) Phase-contrast images of cells at different time points following MNNG exposure. (c) Immunoblot analysis of PARP-1. As a positive control for PARP-1 cleavage, HEK293 cells were treated with 100 µM VP-16 for 24 hours. Actin was used as an internal control.

To better define the type of cell death induced by MNNG, and determine whether necrosis would be secondary to earlier apoptotic events, we determined cell distribution in each quadrant by flow cytometry at different time points after MNNG treatment and Annexin V/PI staining (Fig. 7a). There was no increase in early apoptosis (AV+/PI- staining) at any time point following MNNG exposure (Fig. 7a). Furthermore, neither membrane blebbing (Fig.7b) nor cleaved PARP-1 (Fig.7c), which are markers of the apoptotic process, were observed during the time course. These observations suggest that in HEK293 cells, MNNG induced a necrotic form of cell death following PARP-1 activation.

### Implication of AMPK on MNNG-induced Cell Death

To further elucidate the role of AMPK on MNNG-induced cell death, we incubated cells with Compound C, an inhibitor of AMPK [36], and measured cell death following MNNG exposure (Fig. S3). We observed that the presence of Compound C, amplifies MNNG-induced cell death as shown by a significant increase in PI positive cells in MNNG + Compound C-treated cells compared to MNNG-treated cells (91.2±0.5% versus 68.2±1.9%, *P*<0.01).

**Figure 8:**
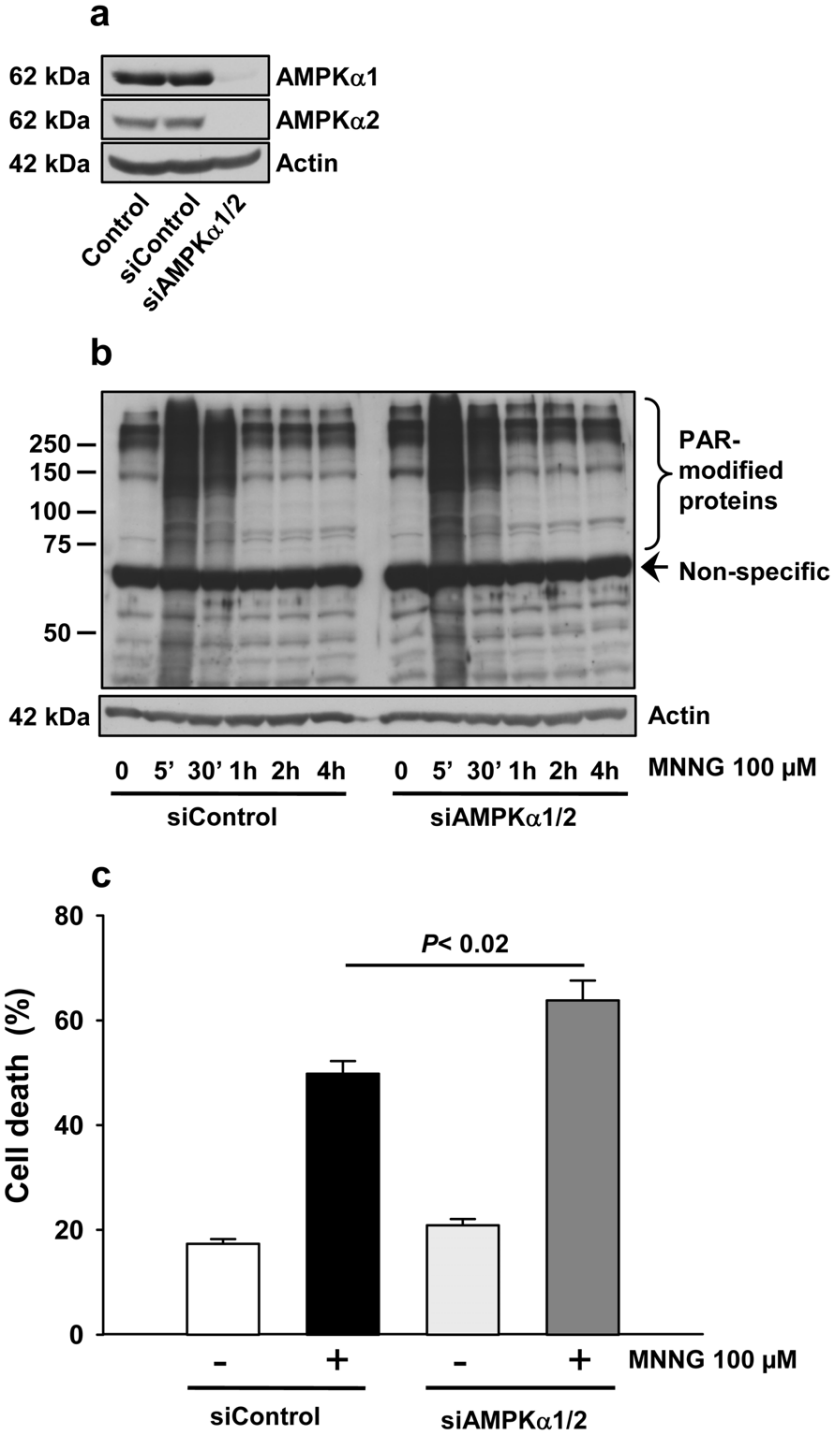
Effect of genetic inhibition of AMPK on PAR synthesis and cell death following MNNG exposure. Cells were transfected with AMPα1 and α2 siRNAs or with control siRNA for 96 h and then treated with 100 µM MNNG. (a) The knock-down of AMPKα1 and AMPKα2 was determined by western blotting. Actin expression was measured as an internal control. (b) PAR accumulation in MNNG treated cells with or without AMPK knock-down was detected by western blot using 96-10 antibody. The blots were also probed with actin antibody to show equal loading. (c) Cell death was evaluated 6 hours after MNNG treatment by staining with PI and Annexin-V-FITC coupled with flow cytometry. Data are presented as the mean ± SEM of three independent experiments.

To further examine the role of AMPK activation on cell death, we blocked this pathway by using siRNA directed against the two catalytic subunits of AMPK: α1 and α2. We observed that a significant decrease in the levels of AMPKα1 and AMPKα2 subunits (Fig. 8a) had no effect on PARP-1 activation as shown by the peak of PAR synthesis 5 minutes following MNNG exposure (Fig. 8b), but significantly increased the number of PI positive cells (63.8±3.8% versus 49.8±2.4% in siAMPKα1/α2 treated cells compared to control siRNA-treated cells, Fig. 8c, *P*<0.02). These results support the hypothesis that AMPK activation following MNNG exposure is a pro-survival signal.

### AMPK Involvement in mTOR Signalling

**Figure 9:**
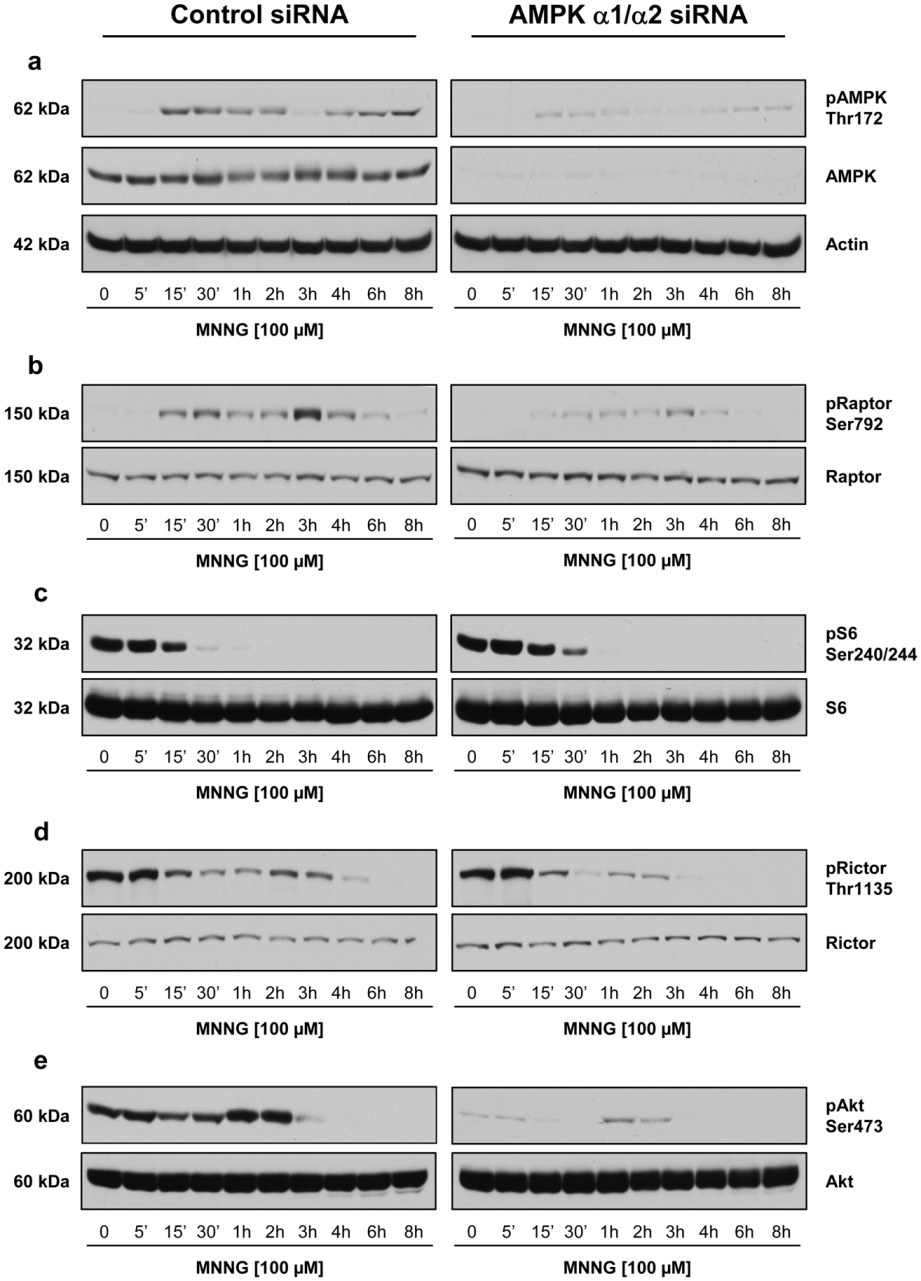
Effect of AMPK knock-down on mTORC1/2 signaling following PARP-1 activation by MNNG exposure. HEK293 cells were treated with siRNA directed against α1 and α2 subunits of AMPK (right panel) or with control siRNA (left panel). Following 100 µM MNNG exposure, cell lysates were analyzed by immunoblotting for (a) AMPK phosphorylation on Thr172, (b) Raptor phosphorylation on Ser792, (c) S6 phosphorylation on Ser240/244, (d) Rictor phosphorylation on Thr1135, and (e) Akt phosphorylation on Ser473. Total protein (Actin, Raptor, S6, Rictor, and Akt) was measured as an internal control (lower panels). In (a), total AMPK was also measured to show the reduced AMPK levels following AMPK knockdown. Immunoblots are representative of three independent experiments.

In order to determine whether the effect of PARP-1 activation on mTOR signaling pathway was mediated by AMPK, we decreased the levels of AMPKα1 and α2 by siRNA and measured the phosphorylation levels of mTORC1 and mTORC2 components following MNNG exposure by immunoblotting analysis. In AMPKα1/α2 siRNA-treated cells, we observed a decreased in the phosphorylation level of AMPK on Thr172 (Fig. 9a and S4a, right panels) and of Raptor on Ser792 (Fig. 9b and S4b, right panels) following MNNG exposure compared to siRNA control cells (Fig. 9a and b, S4a and b, left panels, *P*<0.001). In AMPKα1/α2 siRNA-treated cells we observed a slightly increased phosphorylation of S6 on Ser240/244 up to 30 minutes following MNNG exposure (Fig. 9c and S4c, *P*<0.006) but no significant change in the phosphorylation of Rictor on Thr1135 (Fig. 9d and S4d) compared to control siRNA-treated cells. Unexpectedly the overall phosphorylation level of Akt on Ser473 was significantly decreased in AMPKα1/α2 siRNA-treated cells (Fig. 9e and S4e, *P*<0.001) and interestingly, the increase observed between one and two hours following MNNG exposure in control siRNA-treated cells (Fig. 9e and S4e, left panels) was abrogated in siRNA-treated cells (Fig. 9e and S4e, right panels). These results indicate that AMPK activation following PARP-1 activation has a protective role but is only partially involved in the modulation of mTOR signalling suggesting that other events following PAR synthesis and degradation affects the mTORC1 and mTORC2 signaling pathways leading to cell death.

## Discussion

In this study, we have shown that massive PAR synthesis following MNNG exposure in HEK293 cells affects mTOR signaling, thus modulating survival pathways, but eventually cause cell death by necrosis. We propose that PAR formation following MNNG exposure initially affects the mTORC1 signaling complex. The increase in AMP levels leads to AMPK activation for up to 8 hours (Fig. 2a). We therefore hypothesize that AMPK activation induced by the increase in the AMP/ATP ratio following PAR synthesis inhibits mTORC1 in an attempt to preserve cellular energy. The balance between survival and death signals is essential to cell fate. In our model, PARP-1 activation is rapid and intense and although at first, in an attempt to survive, activation of AMPK and inhibition of mTORC1 occur, the detrimental effects of free ADP-ribose polymers, free ADP-ribose and NAD^+^ depletion overcome the beneficial effect of AMPK activation, and HEK293 cells die by necrosis. Furthermore, our results suggest that ROS production contributes to PARP-1 activation and cell death following MNNG exposure, in accordance with [35]. In fact, in presence of the antioxidant NAC, no PAR synthesis was observed (Fig. 5a), the mTORC1 and mTORC2 complexes modulation was prevented (Fig. 5 and S2) and finally the increase in PI positive cells was abrogated following MNNG exposure (Fig. 6b).

For over 10 years, the role of PARP-1 in the regulation of cell survival and cell death in response to DNA damage has been controversial [3], [17], [18], [23], [37]–[39]. Our studies demonstrate that PARP-1 activation may trigger apoptotic or necrotic responses, depending largely on cell type, functional signaling pathways and stimulus. We have previously shown that in HeLa cells, MNNG activation of PARP-1 results in an AIF-dependent cell death preceded by a nearly complete loss of phosphorylated ERK1/2 [3]. In HEK293 cells treated with the same alkylating agent, we rather observed a necrotic response (Fig. 7). After 6 hours of MNNG exposure, the majority of cells were PI-positive and no apoptotic feature such as membrane blebbing (Fig. 7b) or PARP-1 cleavage (Fig. 7c) was observed. In HeLa cells, which are deficient in LKB1 [40], we observed an accumulation of AMP following MNNG exposure (Fig. 3b) but without significant activation of AMPK (Fig. 3c) or of its substrate, Raptor (Fig. 3d). AMPK can be activated by two distinct signals, an AMP-dependent pathway, mediated by LKB1, and a Ca^2+^-dependent pathway, mediated by CaMKKβ (Ca^2+^/calmodulin-dependent protein kinase kinaseβ) [41]. In HEK293 cells, AMPK is activated by the AMP-dependent pathway mediated by LKB1. It has been shown that intracellular calcium levels increase following MNNG exposure [35] such that in HeLa cells, we hypothesize that the small increase in pAMPK is via a Ca^2+^-dependent pathway, mediated by CaMKKβ. However, the small and short activation of AMPK did not affect the mTORC1 complex as shown by the lack of phosphorylation of Raptor following MNNG exposure in HeLa cells. A recent study [4] addressed the role of AMP production in mitochondrial failure following PARP-1 hyperactivation in HeLa cells. It was shown that degradation of PAR by PARG and NUDIX hydrolases leads to accumulation of AMP, which inhibits the ADP/ATP translocator, thus causing mitochondrial energy failure. The Formentini study and the results presented here suggest new and important effects of AMP accumulation following MNNG-triggered PARP-1 activation. In the LKB1-proficient HEK293 cells, the increased cell death observed in presence of the AMPK inhibitor, Compound C (Fig. S3), and following the knock-down of AMPK (Fig. 8c) suggest a dual role of AMP. Following PARP-1 activation and massive PAR synthesis, the increase in AMP would lead to AMPK activation in an attempt to protect the cell but at the same time could interfere with ATP production and extrusion.

It has been shown that phosphorylation of Raptor by AMPK induces a metabolic check-point to inhibit cell growth [28]. Activation of AMPK and subsequent suppression of mTORC1 activity can also induce a cytoprotective autophagic response [42]–[44]. In our model, we observed AMPK activation and Raptor phosphorylation following PARP-1 activation (Fig. 2a and b). However, even though cells are autophagic competent as shown by the accumulation of LC3-II in presence of HCQ (Fig. 4c and d), it did not lead to induction of autophagy. In fact, no additive effect on autophagic flux was observed as shown by the absence of a significant increase in LC3-II accumulation when MNNG is present (Fig. 4c, and d). In addition, the lack of formation of a punctuate signal of GFP-LC3 transiently transfected in HEK293 cells (Fig. 4b), and the unaltered beclin expression (Fig. 4a) indicate that autophagy is not induced. In summary, in the LKB1-proficient cell line HEK293, MNNG exposure leads to PAR synthesis and energy depletion. The low-energy status promotes AMPK activation and mTORC1 inhibition but surprisingly, does not trigger autophagy. The mechanism leading to mTORC1 inhibition and cell death by necrosis in the LKB1-proficient cell line HEK293 remains to be characterized, but is dependent on PARP-1 activation since all those events are prevented by the PARP inhibitor AG14361.

A feedback loop between the mTORC1/S6K and the PI3K/Akt axis has been described [45]–[47]. In our model, we observed a transient activation of the Akt pathway due to inhibition of the mTORC1/S6K1 axis as shown by a transient increase in Akt phosphorylation on Ser473 (Fig. 2e) when S6 phosphorylation on Ser240/244 decreased (Fig. 2c). Then, Akt phosphorylation is completely lost suggesting that the mTORC2 signaling pathway might also be affected by PARP-1 activation following MNNG exposure. Rictor has been identified as an essential component of the mTORC2 complex which is required for phosphorylation of Akt on Ser473 [14]. It has been recently shown that Rictor is a direct target of the ribosomal S6 kinase-1 [48], [49] and that Rictor phosphorylation at Thr1135 is not critical in the mTORC2 integrity [50] but negatively regulates mTORC2-directed Akt activation and signaling in parallel with other mTORC1 feedback mechanism [49], [51]. The decrease in Rictor phosphorylation on Thr1135 observed following MNNG exposure (Fig. 2d and S1d) coincides with the activation of AMPK (Fig. 2a and S1a). However, the decrease of AMPKα1/α2 subunits by siRNA treatment has no significant effect on the phosphorylation status of Rictor (Fig. 9d and S4d) but significantly affects the phosphorylation of Akt (Fig. 9e and S4e).

In a recent study from our laboratory, Rictor has been identified as a putative PAR-binding protein [52]. Following MNNG exposure, it is conceivable that PAR could bind Rictor and interfere with Rictor phosphorylation. This in turn would lead to inactivation of the mTOR-Rictor complex and loss of Akt phosphorylation on Ser473. Further studies will be needed to evaluate the effects of PAR synthesis on mTORC2 complex formation and its kinase activity. The results obtained with the knock-down of AMPK indicate that AMPK activation is not involved in the modulation of Rictor phosphorylation on Thr1135 following MNNG but affects Akt phosphorylation on Ser473 through a yet unknown mechanism. However, the decrease in Akt phosphorylation observed by 4 hours following MNNG exposure (Fig. 2e) appears to be a consequence of PARP-1 activation and PAR synthesis rather than the trigger of cell death. In fact, the PI3-kinase inhibitor LY294002 only slightly decreased the protective effect of PARP inhibition after MNNG exposure even though Akt phosphorylation was abrogated (data not shown).

Unexpectedly, following AMPK knock-down, S6 phosphorylation is not completely restored (Fig. 9c) despite the fact that AMPK activation and Raptor phosphorylation are abrogated (Fig. 9a and b). P70S6K is controlled by multiple signaling pathways and phosphorylates S6 ribosomal protein. The RAF-MEK-ERK/MAPK pathway is implicated in the phosphorylation and regulation of P70S6K [53], [54]. We have previously shown a decrease in ERK1/2 phosphorylation in HeLa cells following MNNG exposure and PARP-1 activation [3]. We also observed a decrease in ERK1/2 phosphorylation following MNNG exposure in HEK293 cells (data not shown). S6 phosphorylation could be affected by the loss of ERK1/2 phosphorylation following MNNG exposure, explaining why S6 phosphorylation is not completely restored when AMPK is silenced.

**Figure 10:**
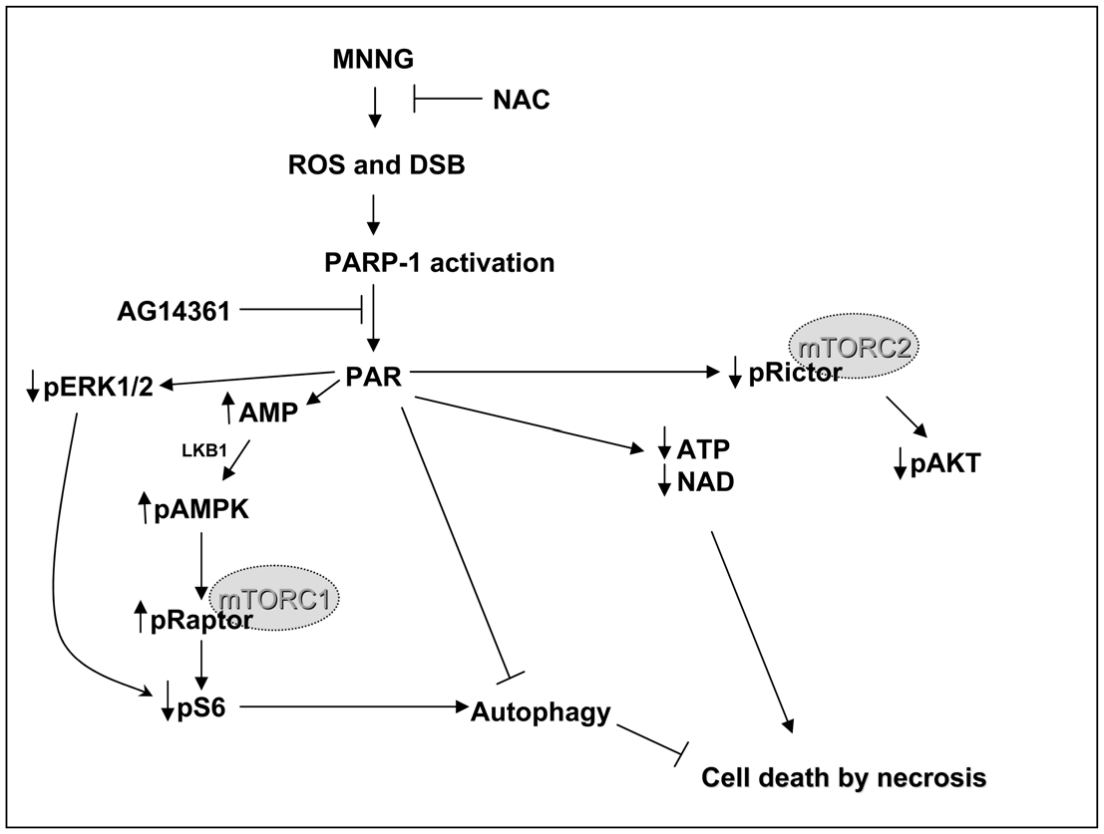
Schematic representation of the possible effects of PARP-1 activation on mTORC1 and mTORC2 components and cell death. Following MNNG exposure, reactive oxygen species (ROS) are produced and DNA double-strand breaks (DSB) are formed. PAR transiently accumulates and its metabolism results in a decrease in ATP and NAD^+^ levels, while cellular AMP increases concomitantly. The high AMP levels activate AMPK by LKB1. AMPK activation causes inactivation of the mTORC1 pathway through phosphorylation of Raptor and inhibition of S6 phosphorylation. S6 phosphorylation may also be affected by the reduced ERK1/2 phosphorylation observed following PAR synthesis. Even though mTORC1 inhibition is the first step towards the establishment of an autophagic state, autophagy is not activated. Rictor phosphorylation is affected by PAR synthesis and Akt phosphorylation at Ser473 is impaired. Finally, in HEK293 cells, PARP-1 activation by MNNG exposure results in necrotic cell death. The PARP inhibitor AG14361 and the antioxidant N-acetyl-L-cysteine (NAC) inhibit PAR synthesis and prevent cell death.

In summary, previous studies from several research teams combined with our novel data reveal that PARP-1 activation triggers a number of signaling pathways resulting in cell death through different mechanisms. Classically, several features are observed following PARP-1 activation leading to cell death: early PAR accumulation and degradation, loss of cellular NAD^+^ and ATP, mitochondrial depolarization, AIF release and translocation to the nucleus, and late caspase activation. Our study reveals a number of novel features triggered by PARP-1 over activation, expanding the wealth of cellular pathways affected by PAR synthesis. Firstly, AMP accumulation resulting from PAR synthesis and degradation triggers AMPK activation and inhibition of the mTORC1 pathway. Secondly, the phosphorylation level of the mTORC2 component, Rictor, is affected by PAR synthesis but not by AMPK activation following MNNG exposure suggesting new and important roles of PAR in these pathways. All these events leading to a necrotic cell death response are summarized in Figure 10. Since PARP-1 activation has been associated with several diseases [55], it is important to understand the signaling mechanisms triggered by PAR.

## Materials and Methods

### Cell Culture

Human embryonic kidney cells, HEK293, from Microbix Biopharmaceuticals (Toronto, ON, Canada) and human cervical carcinoma HeLa cells from ATCC (Manassas, VA) were cultured in Dulbecco’s modified Eagle’s medium (DMEM) supplemented with 10% fetal bovine serum (FBS), 2 mM L-glutamine, 100 U/ml penicillin, and 100 µg/ml streptomycin in a humidified atmosphere of 5% CO_2_ at 37°C. For MNNG treatment, cells were incubated in media containing 100 µM MNNG. Cells were harvested at indicated time points following MNNG exposure. For MNNG experiments longer than one hour, the treatment medium was replaced by fresh medium devoid of MNNG, and cells were cultured for the indicated period of time. For AG14361, NAC, and HCQ treatments, cells were exposed to 5 µM AG14361, 10 mM NAC, or 8 µM HCQ one hour before MNNG addition and remained present throughout the time-course. For Compound C treatment, cells were exposed to 10 µM Compound C 16 hours before MNNG addition. For H_2_O_2_ and VP-16 treatments, HEK293 cells were exposed to 1 mM H_2_O_2_ for 48 hours and 100 µM VP-16 for 24 hours. All treatments were conducted in media supplemented with 5% FBS.

### Reagents and Antibodies

MNNG was purchased from Anachemia (Montreal, QC, Canada). AG14361 was kindly provided by Pfizer (New York, NY). FITC-conjugated Annexin-V and propidium iodide (PI) were from Molecular Probes (Eugene, OR). NAC was from Axxora (San Diego, CA). Compound C was from EMD Biosciences (San Diego, CA). Cell culture reagents were obtained from Invitrogen (Burlington, ON, Canada). All other reagents were from Sigma (Oakville, ON, Canada). Anti-phospho-AMPKα (Thr172), anti-AMPKα, anti-phospho-Raptor (Ser792), anti-Raptor, anti-phospho-Rictor (Thr1135), anti-Rictor, anti-phospho-Akt (Ser473), anti-Akt, anti-phospho-S6 ribosomal protein (Ser240/244), anti-S6 ribosomal protein, anti-Beclin 1, and anti-LC3B antibodies were purchased from Cell Signaling Technology (Beverly, MA). Anti-actin was from EMD Biosciences (San Diego, CA). The GFP-LC3 clone was from GeneCopoeia (Germantown, MD). Anti-PAR (LP96-10) and anti-PARP-1 (clone C2-10) were produced in house [56], [57].

### Western Blot Analysis

After treatments, cells were scraped in 2X Laemmli SDS sample buffer containing 2.5 mM sodium pyrophosphate, 1 mM sodium orthovanadate, 10 mM sodium fluoride, and 5% (v/v) β-mercaptoethanol. Floating cells were previously collected, pelleted by low-speed centrifugation, and combined with the corresponding cell extract. Extracts were sonicated 10 seconds on ice and incubated for 5 minutes at 100°C. Aliquots of the protein extracts were resolved using SDS-PAGE and transferred to polyvinylidene-fluoride (PVDF) membranes (Millipore, Bedford, MA). After incubating 1 hour with TBS-MT (Tris-buffered saline (TBS) with 0.1% (v/v) Tween-20 (TBS-T) containing 5% non-fat milk), the membrane was probed with primary antibodies diluted in TBS-MT overnight at room temperature with shaking. After washing with TBS-T, species-specific horseradish peroxidase-conjugated secondary antibody (Jackson ImmunoResearch Laboratories, West Grove, PA) was added for 1 hour at room temperature. Signals were detected with Western Lightning™ Chemiluminescence Reagent Plus kit (Perkin Elmer, Boston, MA).

### Detection of Cell Death by Flow Cytometry Assay

From 1 to 6 hours after MNNG addition, floating and attached cells were collected together, pelleted by low-speed centrifugation, washed with ice-cold phosphate buffered saline (PBS) and resuspended in Annexin V-binding buffer (10 mM HEPES (pH 7.4), 140 mM NaCl and 2.5 mM CaCl_2_). To detect cell death, cells were incubated with FITC-conjugated Annexin-V and 1 µg/ml PI for 30 minutes in the dark on ice. Cells were analyzed immediately after incubation using a FACScan Beckman-Coulter Epics XL flow cytometer.

### Transient Transfection of Small Interfering RNAs

The small interfering RNA (siRNAs) sequences to silence the human α1/α2 AMPK isoforms and the negative control siRNA were from Invitrogen. The siRNA sequences for AMPKα1 were forward 5′-CCC AUC CUG AAA GAG UAC CAU UCU U-3′ and reverse 5′-AAG AAU GGU ACU CUU UCA GGA UGG G-3′, and AMPKα2 forward 5′-ACC GAG CUA UGA AGC AGC UGG AUU U-3′ and reverse 5′-AAA UCC AGC UGC UUC AUA GCU CGG U-3′. HEK293 cells were transfected with 20 nM of the siRNAs using HiPerfect (Qiagen, Toronto, ON, Canada) according to the supplier’s protocol. Cells were used for experiments 96 hours following transfection.

### Transient Transfection and Microscopy Analysis

Cells were grown on coverslips in a 35-mm culture dish and transfected with 0.5 µg of the GFP-LC3 expression plasmid using Effectene (Qiagen) according to the supplier’s protocol. After treatment, as defined in the figure legends, cells were washed with PBS and fixed with 4% formaldehyde in PBS for 15 minutes at room temperature. Nuclei were stained with Hoechst 33242 solution (Sigma). Coverslips were inverted on a drop of Fluoromount-G (Southern Biotech, Birmingham, AL). Transfected cells were observed and images were taken using a fluorescence microscope (Nikon, E1000).

### NAD^+^, AMP, and ATP Measurements

The nucleotides were extracted from HEK293 cells cultured in six-well dishes following MNNG exposure as previously described [58]. Briefly, culture medium was removed, cells were washed with ice-cold PBS, and 500 µl of ice-cold 0.4 M perchloric acid was added. The culture dishes were cooled to −80°C then cell lysates were thawed on ice, scraped off, and transferred to microfuge tubes. Samples were centrifuged at 15,000 g for 10 minutes at 4°C. The supernatant was neutralized with 50 µl 4 M K_2_CO_3_, kept on ice for 10 minutes, and placed at −80°C for 1–2 hours to allow precipitation of the perchlorate. Samples were centrifuged again and supernatants were kept at −80°C until nucleotide measurements.

### AMP Determination by HPLC

For the quantification of AMP present in HEK293 cell extracts, the nucleotides were separated by HPLC using a SynChropak AX100 anion exchange column (Phenomenex, 250×4.6 mm i.d.). The nucleotides were eluted isocratically at ambient temperature with a mobile phase of 125 mM K_2_HPO_4_, 0.5 M KCl, adjusted to pH 6 with 5N NaOH. Forty µl of nucleotide cell extracts described above were diluted to 100 µl in the mobile phase and 50 µl were injected into the instrument. The pumps were adjusted to a 1.0 ml/min flow rate. Absorbance was monitored at 260 nm and retention time for AMP was 7.3 minutes. The amount of AMP contained in each fraction was measured by comparing the height of the peak with a standard curve of AMP, ranging from 0 to 20 pmoles.

### ATP Determination by Fluorometric Assay

ATP quantification was performed using the Biovision ATP Colorimetric/Fluorometric Assay Kit (Cedarlane, Burlington, ON, Canada). Briefly, the fluorometric assay was performed as described by the manufacturer, using a standard curve of 0 to 200 pmoles. Ten µl of nucleotide extract, diluted to 50 µl with ATP Assay Buffer, was used for each quantification. Plates were incubated in the dark for 30 minutes. Data was acquired on a Cytofluor^tm^ 2350 (Millipore Co., Bedford, MA) using black 96-well assay plates. ATP was quantified by comparing the absorbance values obtained at 590 nm with those of the standard curve.

### NAD^+^ Quantification by Colorimetric Assay

NAD^+^ was measured using a colorimetric assay as described by Shah G.M. *et al.* [59] with slight modifications. The assay was carried out in a 96-well flat-bottom plate. The reaction mixture consisted of 5 volumes of NAD^+^ premix (to obtain final concentrations of 114 mM bicine (pH 7.8), 570 mM ethanol, 0.48 mM MTT, 4.8 mM EDTA, 1 mg/mL BSA), one volume of the activation solution (1 mg/ml alcohol dehydrogenase in 0.1 M bicine (pH 7.8) and one volume of 40 mM phenazine ethosulfate). The nucleotide extract (10 µl) was mixed with 90 µl distilled water and 50 µl freshly prepared reaction mix. The colour was developed in the dark for 1 hour at room temperature and the absorbance was monitored at 600 nm in an EAR 400AT Easy Reader microplate reader (SLT Labinstruments, Vienna, Austria). A standard curve of 0 to 20 pmoles of NAD^+^ was also prepared for quantification.

### Statistical analysis

Results are expressed as mean ± standard error of the mean (SEM). Differences between groups were assessed using one-way or two-way analysis of variance (ANOVA) followed by Student-Newman-Keuls multiple comparison or by Student’s *t*-test. *P* values below 0.05 were considered significant.

## Supporting Information

Figure S1 **Densitometry values of the phosphorylated protein normalized to the total protein quantified using Gene Tools software from Perkin Elmer in MNNG-treated cells alone (left panel) or in combination with AG14361 (right panel).** (a) pAMPK/AMPK ratio, (b) pRaptor/Raptor ratio, (c) pS6/S6 ratio, (d) pRictor/Rictor ratio, (e) pAkt/Akt ratio. Data are presented as the mean ± SEM of three to four independent experiments. *P<0.05, **P<0.01, ***P<0.001 compared to control (0).
(TIF)

Figure S2 **Densitometry values of the phosphorylated protein normalized to the total protein quantified using Gene Tools software from Perkin Elmer in MNNG-treated cells alone (left panel) or in combination with NAC (right panel).** (a) pAMPK/AMPK ratio, (b) pRaptor/Raptor ratio, (c) pS6/S6 ratio, (d) pRictor/Rictor ratio, (e) pAkt/Akt ratio. Data are presented as the mean ± SEM of three to four independent experiments. *P<0.05, **P<0.01 compared to control (0).
(TIF)

Figure S3 **Effect of the AMPK inhibitor, Compound C, on MNNG-induced cell death.** HEK293 cells were treated with MNNG alone or in combination with 10 µM Compound C sixteen hours prior to MNNG exposure. Cell death was evaluated as the percentage of PI positive cells 6 hours after MNNG treatment by staining with PI and Annexin-V-FITC coupled with flow cytometry. Data are presented as the mean ± SEM of two independent experiments.
(TIF)

Figure S4 **Densitometry values of the phosphorylated protein normalized to the total protein quantified using Gene Tools software from Perkin Elmer following MNNG exposure of cells treated with control siRNA (left panel) or with siRNA against AMPK α1/α2 (right panel).** (a) pAMPK/Actin ratio, (b) pRaptor/Raptor ratio, (c) pS6/S6 ratio, (d) pRictor/Rictor ratio, (e) pAkt/Akt ratio. Data are presented as the mean ± SEM of three independent experiments. Differences between groups were assessed using two-way analysis of variance.
(TIF)
