# Supplementary figures and images for: PARP-1 Modulation of mTOR Signaling in Response to a DNA Alkylating Agent
Source: PLoS One. 2012 Oct 24;7(10):e47978. doi: 10.1371/journal.pone.0047978 (PMC3480502; doi:10.1371/journal.pone.0047978)

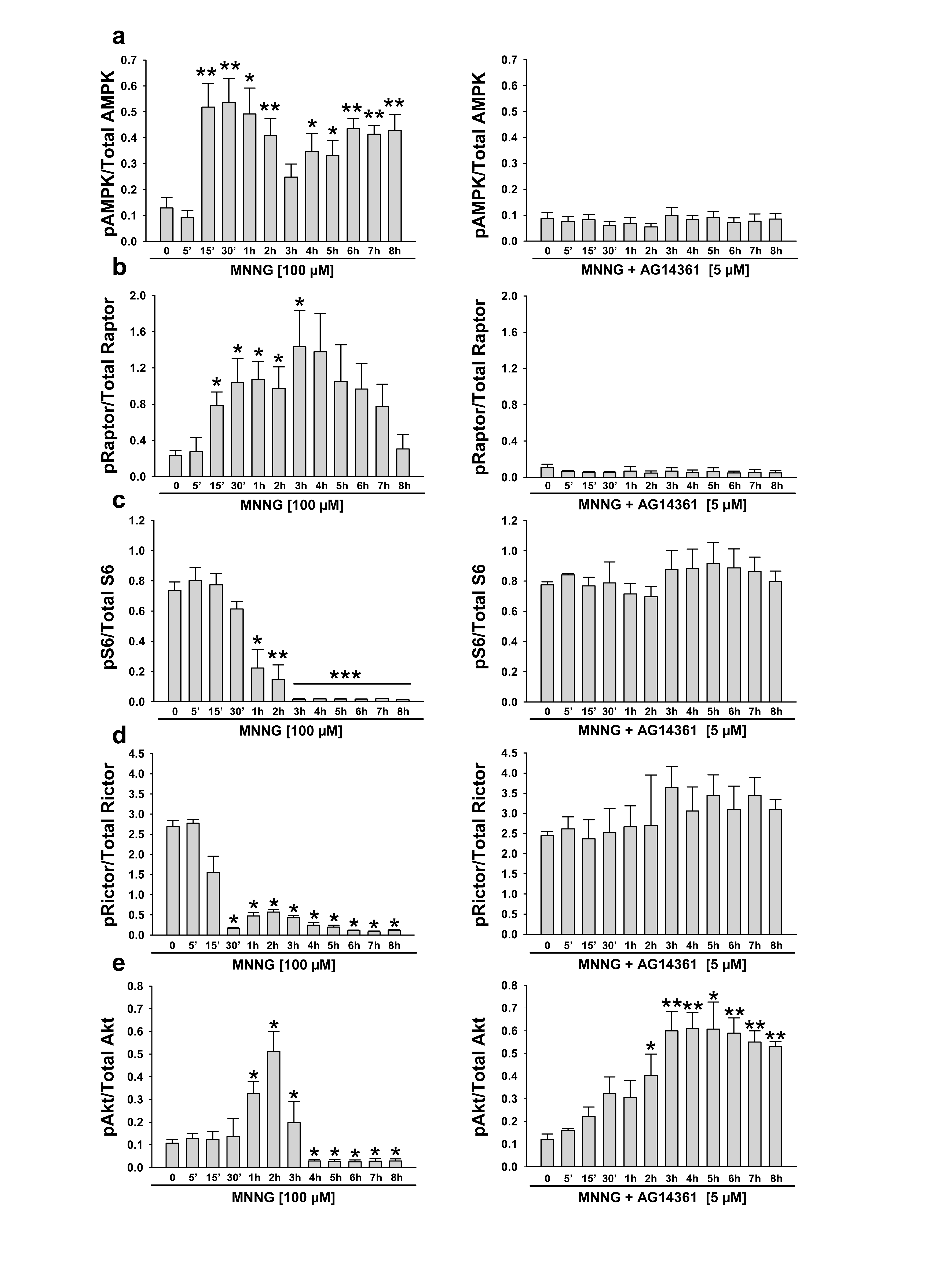

Supplement: Figure S1 — Densitometry values of the phosphorylated protein normalized to the total protein quantified using Gene Tools software from Perkin Elmer in MNNG-treated cells alone (left panel) or in combination with AG14361 (right panel). (a) pAMPK/AMPK ratio, (b) pRaptor/Raptor ratio, (c) pS6/S6 ratio, (d) pRictor/Rictor ratio, (e) pAkt/Akt ratio. Data are presented as the mean ± SEM of three to four independent experiments. *P<0.05, **P<0.01, ***P<0.001 compared to control (0). (TIF) [file pone.0047978.s001.tif]

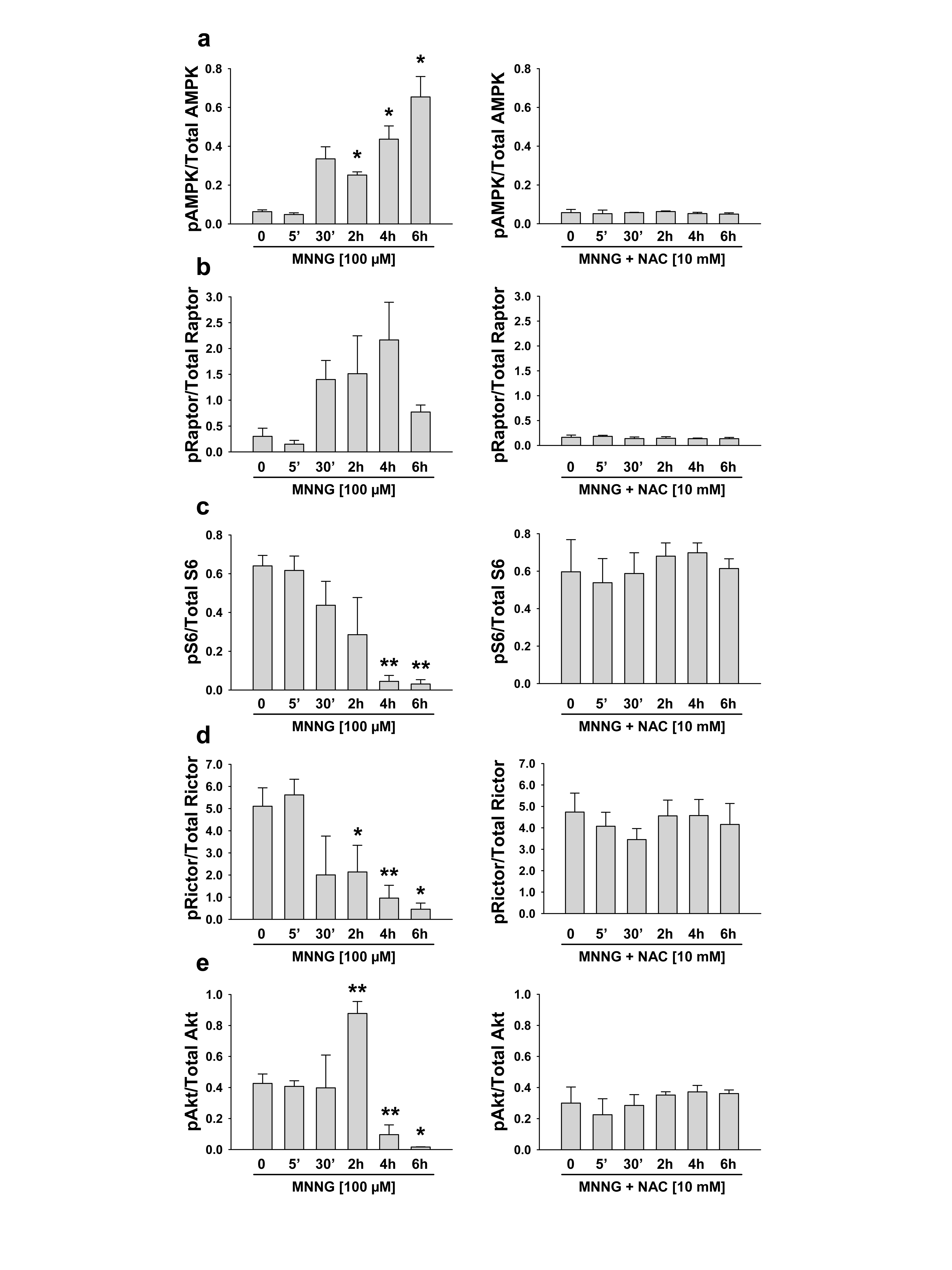

Supplement: Figure S2 — Densitometry values of the phosphorylated protein normalized to the total protein quantified using Gene Tools software from Perkin Elmer in MNNG-treated cells alone (left panel) or in combination with NAC (right panel). (a) pAMPK/AMPK ratio, (b) pRaptor/Raptor ratio, (c) pS6/S6 ratio, (d) pRictor/Rictor ratio, (e) pAkt/Akt ratio. Data are presented as the mean ± SEM of three to four independent experiments. *P<0.05, **P<0.01 compared to control (0). (TIF) [file pone.0047978.s002.tif]

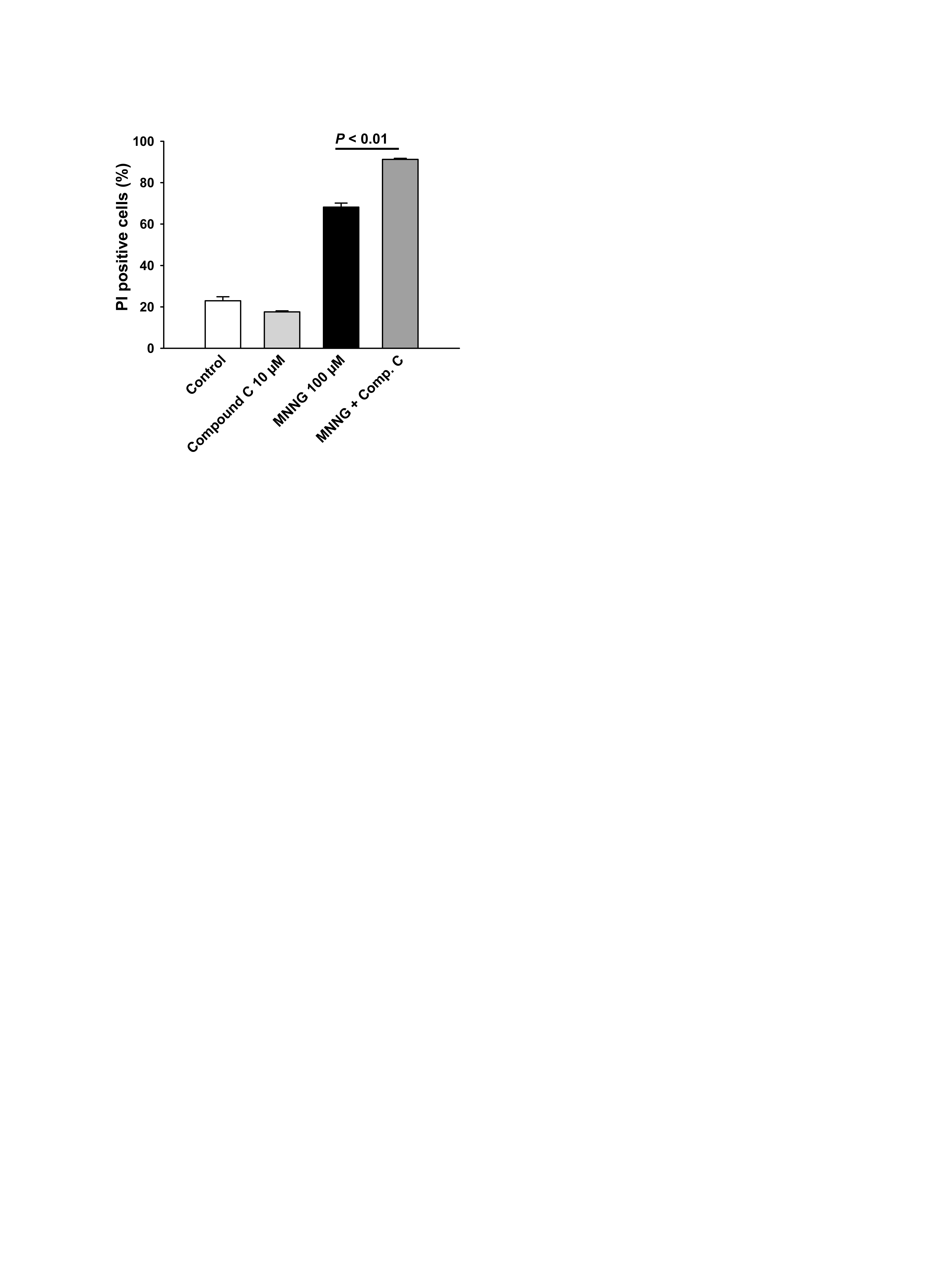

Supplement: Figure S3 — Effect of the AMPK inhibitor, Compound C, on MNNG-induced cell death. HEK293 cells were treated with MNNG alone or in combination with 10 µM Compound C sixteen hours prior to MNNG exposure. Cell death was evaluated as the percentage of PI positive cells 6 hours after MNNG treatment by staining with PI and Annexin-V-FITC coupled with flow cytometry. Data are presented as the mean ± SEM of two independent experiments. (TIF) [file pone.0047978.s003.tif]

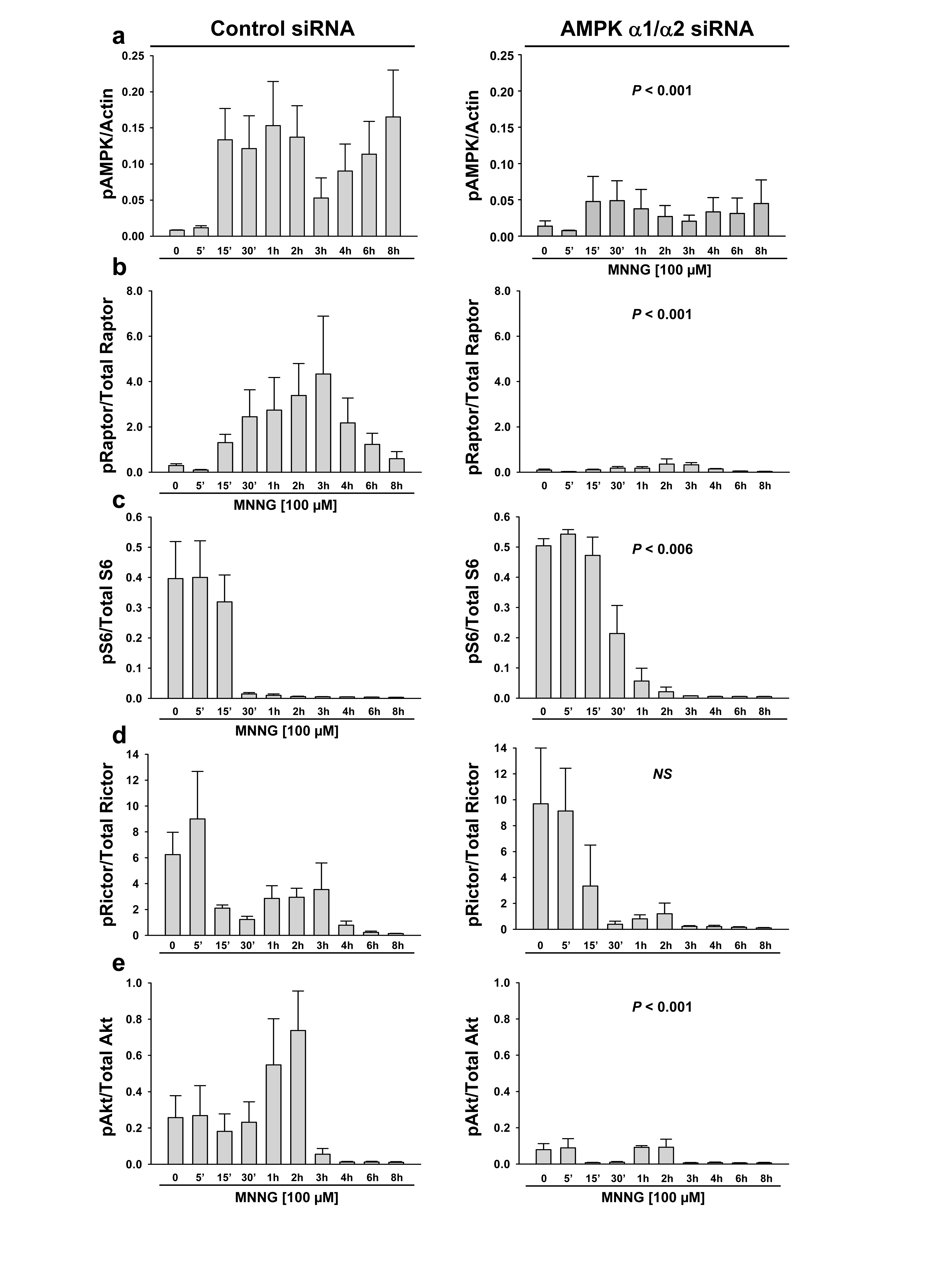

Supplement: Figure S4 — Densitometry values of the phosphorylated protein normalized to the total protein quantified using Gene Tools software from Perkin Elmer following MNNG exposure of cells treated with control siRNA (left panel) or with siRNA against AMPK α1/α2 (right panel). (a) pAMPK/Actin ratio, (b) pRaptor/Raptor ratio, (c) pS6/S6 ratio, (d) pRictor/Rictor ratio, (e) pAkt/Akt ratio. Data are presented as the mean ± SEM of three independent experiments. Differences between groups were assessed using two-way analysis of variance. (TIF) [file pone.0047978.s004.tif]
